# Physical Activity and Physical Fitness of School-Aged Children and Youth with Autism Spectrum Disorders

**DOI:** 10.1155/2014/312163

**Published:** 2014-09-16

**Authors:** Kiley Tyler, Megan MacDonald, Kristi Menear

**Affiliations:** ^1^College of Public Health and Human Sciences, Oregon State University, Corvallis, OR 97331, USA; ^2^School of Education, University of Alabama at Birmingham, Birmingham, AL 35294, USA

## Abstract

Autism spectrum disorder (ASD) is characterized by impairments in social communication deficits and the presence of restricted and repetitive behaviors, interests, or activities. Literature comparing the physical activity and fitness of children with ASD to typically developing peers is in need of attention. The purpose of this investigation was to examine the physical activity and fitness of school-aged children with ASD (*N* = 17) in comparison to typically developing peers (*N* = 12). Participants with ASD completed diagnostic and developmental assessments and a series of physical fitness assessments: 20-meter multistage shuttle, sit-and-reach test, handgrip strength, and body mass index. Physical activity was measured using accelerometry and preestablished cut-points of physical activity (Freedson et al., 2005). MANCOVA revealed significant between-group effects in strength (*P* = .03), while ANCOVA revealed significant between-group effects in sedentary (*P* = .00), light (*P* = .00), moderate (*P* = .00), and total moderate-to-vigorous (*P* = .01) physical activity. Children with ASD are less physically active and fit than typically developing peers. Adapted physical activity programs are one avenue with intervention potential to combat these lower levels of physical activity and fitness found in children with ASD.

## 1. Introduction

As the incidence rate of autism spectrum disorder (ASD) increases, so does the interest in the health and wellness of these individuals. Similar to their typically developing peers, youth with ASD face increased rates of obesity [1, 2] and decreased engagement in physical activities [3]. Reviews of physical activity patterns indicated that older adolescents with ASD are less physically active than their younger peers with ASD [4–6]. The contributing factors to the obesity and physical inactivity epidemic in this population may be both diagnosis-specific and environmental [7].

Profiles of motor skills and physical fitness in children and youth with ASD have documented delayed performance levels. Pan [8] assessed 62 adolescent males with (*n* = 31) and without (*n* = 31) ASD aged 10–17 years using the Bruininks-Oseretsky Test of Motor Proficiency (2nd edition), the BROCKPORT Physical Fitness Test, and the bioelectrical impedance analysis. Results indicated that adolescents with ASD had significantly lower scores on all motor proficiency and fitness measures, except body composition, than adolescents without ASD and that the types of associations between the two measures differed significantly across the groups. Staples and Reid [9] compared the fundamental movement skills of 25 children and youth with ASD (ages 9–12 years) to three typically developing groups of children using the Test of Gross Motor Development (TGMD-2). Their results suggested that children and youth with ASD experience more than just delays in movement abilities but that movement deficits in motor skill performance existed.

Literature that compares the physical activity and fitness of children and youth with ASD to their typically developing peers is scarce, but what does exist uses common fitness measures such as the shuttle run, sit-and-reach, and handgrip dynamometer [8] and activity measures such as accelerometry [3]. Health-related physical fitness, which is embedded in physical activity through physical exercise, consists of body composition, aerobic fitness (aerobic capacity, power, and endurance), flexibility, and muscular fitness (muscular strength and muscular endurance) [10–12]. Our study includes these physical fitness measures, in addition to a physical activity measure. The indicated measures are used to describe the physical activity and physical fitness of school-aged children with ASD in comparison to typically developing peers. By using a similar age range to the studies cited above, including both male and female participants with a range of severity (as indicated through calibrated autism severity scores), and adding additional measures of physical fitness compared to other studies, our study provides further evidence of the profiles of this population compared to their typically developed peers as it relates to physical activity and physical fitness. Thus, the purpose of this study is to determine the physical fitness and physical activity of children with ASD, compared to their typically developed peers.

## 2. Method 

### 2.1. Participants

Children and youth with ASD (*N* = 17,  M = 9,  F = 8) and without ASD (*N* = 12,  M = 6,  F = 6) between the ages of 9 and 18 years were recruited for this study. Recruitment occurred through the dissemination of study flyers, autism-based programs, local schools, local rehabilitation facilities, and word of mouth. Participants were not excluded based on race/ethnicity or gender.

### 2.2. Procedure

Each parent/caregiver of each participant completed a supplemental questionnaire, which included demographic information such as age of participant, maternal education, income, ethnicity, and diagnosis of an ASD. Data collection took place in a lab-based setting, where participants completed the majority of assessments over the course of two days. Each assessment session took into consideration the comfort and scheduling needs of each participant and family. The order of assessments typically consisted of the diagnostic assessment and developmental assessment, followed by a series of physical fitness assessments in aerobic fitness, muscular strength, flexibility, and anthropometric measures (height and weight). Physical activity was measured through accelerometry. The assessments are described below.

#### 2.2.1. Diagnostic Assessment

Participants with ASD, per parental report, were administered the autism diagnostic observation schedule (ADOS) [13–15] by a trained research administrator. The senior investigator on this project was research trained and reliable on ADOS administration. Each member of the research team attained interrater reliability of ADOS administration exceeding 80% exact agreement over the course of three consecutive administrations before the study began with the senior investigator on this project. Thus, within site reliability was obtained. The ADOS is a semistructured standardized assessment of communication, social interaction, and play for individuals with autism spectrum disorder or suspected of having autism. This diagnostic tool is commonly used in practice and research and is considered the “gold standard” in terms of standardized diagnostic assessments [16]. Calibrated severity scores [13] were used in this study to confirm diagnosis; these scores are consistent with ADOS-2 criteria [13].

#### 2.2.2. Developmental Assessment

The Stanford-Binet Intelligence Scale Fifth Edition (SB5) was administered to assess cognitive abilities of each participant [17]. The SB5 is an individually administered assessment of cognitive abilities for individual of 2–85 years. If the participant was unable to progress through the SB5, typically based on limited language, the Differential Ability Scale Second Edition (DAS-II) was administered. The DAS-II is a standardized comprehensive assessment of cognitive abilities [18]. The DAS-II consists of a variety of subtests including verbal and visual working memory, immediate and delayed recall, visual recognition and matching, process and naming speed, phonological processing, and understanding of basic number concepts [18]. Ratio verbal and nonverbal IQ scores were calculated from the SB-5 and the DAS-II, allowing for comparison of verbal and nonverbal abilities across tests. Ratio nonverbal IQ was calculated by dividing the average age equivalent across nonverbal subtest scales, by chronological age in months, and then multiplying by 100. Ratio verbal IQ was calculated in the same manner using the verbal subtest.

#### 2.2.3. Aerobic Fitness Assessment

Aerobic fitness was assessed using the 20-meter multistage shuttle run [19]. The 20-meter multistage shuttle run is a valid and reliable assessment of aerobic power in children [19], with good test-retest reliability for children (*r* = .89,  *P* = .05). Each participant was provided with a shuttle run demonstration by the examiner as well as a practice trial before the trials began. During each measured trial, an undergraduate student served as the participant's running partner in an effort to help with the pacing of each shuttle run. The procedures for the 20-meter multistage shuttle run include the participant running back and forth between two sets of cones (shuttles) spaced 20 meters apart. The rate at which the participant runs is controlled by an audio recording that “beeps” when the participant should reach the opposing set of cones. The beeps are equally spaced within one-minute “stages,” starting slowly and accelerating incrementally every minute thereafter. If the participant was unable to complete two consecutive stages in accordance with the prompted “beeps,” the test was terminated and the final distance was recorded. The velocity of the final shuttle completed by the individual was used to determine the estimated maximal aerobic power (VO_2max⁡_) for that individual [19]. The 20-meter multistage shuttle was converted into VO_2max⁡_ (*Y*, mL kg^−1^ min^−1^) values. These values were obtained from the maximal shuttle run speed (*Xlt* km h^−1^) and age (*X*2, year rounded to the lower integer) for children between the ages of 6 and 18 years (*y* = 31.025 + 3.238*X*1 − 3.248*X*′2 + 0.1536*X* ^*r*^1*X*′2) [19].

#### 2.2.4. Flexibility Assessment

Flexibility was assessed using the sit-and-reach test [20]. The sit-and-reach test is a valid and reliable measure used to determine trunk and lower body flexibility in children [21]. Standardized protocols were followed [20] in addition to several bouts of verbal and nonverbal direction (i.e., reminders to keep the legs straight during the stretch forward). Each participant was initially shown how to perform the sit-and-reach test by the examiner and was provided a practice trial before the trials began.

#### 2.2.5. Strength Assessment

Strength was measured using a handgrip strength assessment. In this test, the participant was asked to stand and grip a Jamar Hydraulic Hand Dynamometer [22] with the dominant hand. The participant forcefully squeezed the dynamometer while holding the arm at 90 degrees of flexion and continued squeezing the instrument while straightening the arm to full flexion. The entire action was performed over the course of three separate trials. Each participant was provided with an initial practice trial and task demonstration. During the three trials, each participant was encouraged to squeeze the instrument to full potential while simultaneously straightening their arm to full flexion. The maximal force production, derived from the average of all three trials, was recorded by the examiner. Handgrip strength is a valid and reliable measure of upper-body muscular strength (*r* = .90) in typically developing children ages of 6–12 years [23].

#### 2.2.6. Physical Activity Assessment

Physical activity was measured using an accelerometer, the ActiGraph GTX3+ (Pensacola, FL), over a seven-day period. This is a valid and reliable measure of physical activity for children with disabilities [24]. All participants were asked to wear the monitor for all waking hours of the day on the right hip using an elastic belt. The monitor was worn for all activities except swimming, showering/bathing, and sleeping and was indicated through self-report logs. The parents/caregivers of the participants were provided with a self-report log to record any times when the monitor was not worn (i.e., forgetting to put it on in the morning, taking it off for comfort, or any other reasons for which it may have been removed). Accelerometry data was compared to parent logs and reduced to physical activity categories of sedentary, light, moderate, and moderate-to-vigorous these were derived from established cut-points [25]. Data were reduced into average time spent in the physical activity categories described. Zero activity counts lasting for a period longer than 5 minutes were considered a nonwear period and therefore excluded from data analysis [26].

#### 2.2.7. Anthropometric Measures

Height and weight of each participant was measured without shoes. Each participant was provided with an initial task demonstration and practice trial if needed. Height was measured in centimeters to the nearest tenth of a millimeter with the portable stadiometer, model: SECA S-214. Weight was measured in kilograms to the nearest gram with the Health O Meter H-349KL digital scale. Each participant's body mass index (BMI) was calculated using the standard formula: body mass (in kilograms) divided by height squared (in meters) [12, 27]. BMI is a valid and reliable (*r* = .90) means of assessing body composition (height in relation to weight) in children [12]. Measurements were taken twice at each site and rounded to the nearest tenth of a millimeter.

### 2.3. Data Analysis

Multivariate analysis of covariance (MANCOVA) was used to determine each physical fitness variable of aerobic fitness, flexibility, strength, and BMI of school-aged participants with an ASD compared to typically developing peers. Analysis of covariance (ANCOVA) was used to determine the physical activity of school-aged participants with an ASD compared to participants without an ASD. Covariates for all analyses included age. IQ was highly correlated with age; thus only age was used in these analyses.

## 3. Results

A total of 17 participants with an ASD (*n* = 9 males and *n* = 8 females) and 12 participants without an ASD (*n* = 6 males and *n* = 6 females) between the ages of 9 and 17 years (mean = 12.6 years) were included in this study. Demographic information is presented in Table 1. There were no relationships between physical fitness and income, maternal education, or ethnicity. Missing values were resolved through single mean substitution [28] by group for the variable of interest. Missing variables of physical fitness accounted for less than 10% of missing data. The most common missing variable was physical activity; less than 20% of data were missing in this variable.

### 3.1. Physical Fitness

MANCOVA was used to determine the physical fitness of school-aged participants with ASD and typically developing peers. Age was used as a covariate in this model because the distribution of age was different across groups. Participants with an ASD had more 15-year-olds than any other age, whereas participants without an ASD had a higher distribution of 9-year-olds. The MANCOVA revealed nonsignificant effects (F = 1.59; *P* = .21) between groups, indicating that no significant differences existed in physical fitness between participants with an ASD and participants without an ASD. Between-group effects reached significance in strength (*P* = .03; F = 3.5), suggesting that participants with ASD have lower strength compared to participants without an ASD. Flexibility, BMI, and VO_2max⁡_ (calculated from the 20-meter multistage shuttle run) did not have a significant result. Physical fitness by group is presented in Table 2.

### 3.2. Physical Activity

ANCOVA resulted in between-group effects in sedentary (*P* = .00), light (*P* = .00), moderate (*P* = .00), and moderate-to-vigorous (MVPA) (*P* = .01) physical activity. Children with ASD spent less time in light, moderate, and moderate-to-vigorous physical activity and spent more time in sedentary behavior when compared to typically developing peers. Physical activity results by group are presented in Table 3.

## 4. Discussion and Future Directions

Children with an ASD are less physically fit in the strength domain and less physically active than their peers without disabilities. Yet physical fitness in flexibility, aerobic fitness (VO_2max⁡_), and BMI demonstrates similar results compared to peers without disabilities. Although these results provide further evidence that children with an ASD face known health disparities, they also indicate that aspects of physical fitness are attainable and comparable to peers without ASD.

Our data indicated that children and youth with an ASD performed the shuttle run at only slightly lower levels than their peers without an ASD. This finding, although discrepant, is encouraging given the potentially negative overall health impact of low cardiovascular endurance. This finding further suggests that children with an ASD are more similar to their peers in this area; thus physical education, community-based programs, and other physical activity opportunities may provide an opportunity to further bridge the gap and health disparity between children with an ASD and their peers without an ASD. Supporting the use of peer mentoring in data collection efforts was an encouraging tool for success (i.e., children with ASD completing the task). Knowledge of how shuttle-run performance, ultimately peak oxygen uptake, associates with gender, BMI, age, height, and weight in children and youth with ASD may further aid in better understanding, where to intervene, and how to improve the fitness of school-aged children with ASD [29].

The finding of lower levels of strength (strength with ASD M = 20.3, F = 14.3, without ASD M = 26.4, F = 15.9) indicated a weak area of physical fitness for children with an ASD. Based on the results of this study, strength is in need of improvement, suggesting a clear starting point for physical fitness-based interventions.

It is important to note the practical implications of this research methodology as it relates to physical education. The physical fitness and physical activity assessments we employed are common approaches used by physical educators. Too often, students with disabilities, and particularly students with an ASD, are left out of physical fitness assessment data collection. Our participants with an ASD were able to complete all assessments as were their peers. Not only does federal special education law (i.e., Individuals with Disabilities Education Act and 20 U.S.C. § 1400) addresses the right of students with disabilities to participate in physical education, but also research indicates that children and youth with an ASD have a higher prevalence of obesity compared to children without an ASD [2, 30]. Additionally, Memari et al. [5] found a substantial reduction in physical activity across the adolescent years in youth with an ASD. Therefore, we encourage parents, teachers, and administrators to include students with an ASD in physical fitness and physical activity assessments and provide them with individualized information about associated behaviors that can impact their health into adulthood.

Our ability to complete physical fitness and physical activity assessments of children and youth with an ASD has additional educational implications. The relationship between academics and physical activity is a current topic of discussion in the United States [31–33] and federal initiatives aimed at improving the fitness of our youth have been implemented nationwide, with health and education in mind (e.g., Let's Move, Healthy People 2020). Eveland-Sayers et al. [34] investigated physical fitness and academic achievement in elementary school children and found a link between specific components of physical fitness and academic achievement. Future research might consider applying a similar approach for students with an ASD.

In this study, participants with ASD were less physically fit, in the strength domain, and less physically active than their peers without disabilities. Similarly, previous research confirms these findings [8, 35]. Our participants met the 2008 Physical Activity Guidelines, set by the U.S. Department of Health and Human Services, of 60 minutes/day of moderate-to-vigorous physical activity but had significantly lower activity time than their peers without disabilities. These results are not tremendously different from those of Bandini et al. [3] who found that children with an ASD and those without an ASD spent a similar amount of time in moderate and vigorous physical activity. Contributing factors to the physical activity health disparity present in children with ASD may be the result of diagnostic and environmental determinants [7]. Yet there are many known determinants of physical inactivity in children; more research is needed on the physical activity determinants specific to children with ASD. In conclusion, our results are encouraging as they indicate that children and youth with an ASD show capacity to meet daily guidelines for physical fitness and activity.

### 4.1. Limitations

Limitations to this study include unequal sample sizes and unmatched controls; however, these limitations were accounted for in analyses. This sample of children with ASD consisted of more females (*N* = 17, males = 9, females = 8) than would be expected given the current ratio 5 males : 1 female [36]. Participants completed all assessment, yet individual accommodations were implemented when needed (i.e., all assessments were performed in a single day or assessments were split over two days based on participant/familial needs). Finally, this study consisted of a small sample size; thus additional studies with larger sample sizes are needed.

## 5. Conclusion

These results provide further evidence that children with an ASD face known health disparities, and efforts to promote physical activity in school and through public health initiatives need to include children and youth with an ASD.

## Figures and Tables

**Table 1:** Participant demographic information.

	Children with autism (*N* = 17)		Children without autism (*N* = 12)		*F* value	*P*
	M ± SD/frequency	Min–max	M ± SD/frequency	Min–max		
Age	12.6 ± 2.3	9–17	9.0 ± 1.8	9–14	1.49	0.23
Ratio verbal IQ	60.1 ± 25.2	16–110	112.3 ± 99.0	45–385	2.20	0.14
Ratio nonverbal IQ	65.7 ± 40.0	16–172	73.1 ± 19.3	49–105	1.42	0.24
CSS	8.6 ± 1.54	4–10				
Weight (kg)	52.0 ± 27.1	24–128	46.2 ± 14.1	31–81	3.81	0.06
Height (cm)	154.4 ± 15.38	128–184	153.9 ± 14.1	134–176	0.35	0.85
Gender	M = 9/F = 7		M = 6/F = 6			
Maternal education ethnicity	4 no college/12 college 12 Caucasian/4 other		2 no college/11 college 10 Caucasian/3 other			

Note: M: mean; SD: standard deviation. *P ≤ 0.05.

**Table 2:** Physical fitness by group.

	Children with autism (*N* = 17)		Children without autism (*N* = 12)		*F* value	*P*
	M ± SD	Min–max	M ± SD	Min–max		
VO_2max_	39.9 ± 5.8	31–57	42.0 ± 5.3	32–53	0.07	0.93
Strength	17.5 ± 8.7	6–38	21.1 ± 11.1	10–40	4.7	0.03∗
Flexibility	23.7 ± 8.3	12–41	31.5 ± 10.0	15–44	3.5	0.06
BMI (kg/m^2^)	20.4 ± 6.6	11–34	19.1 ± 4.3	14–30	0.30	0.58

*P ≥ 0.05.

**Table 3:** Daily average time spent in physical activity by group.

	Children with autism (*N* = 17)		Children without autism (*N* = 12)		*F* value	*P*
	M ± SD	Min–max	M ± SD	Min–max		
Sedentary	452.1 ± 100.5∗∗∗	218–572	369.14 ± 105.7	143–518	13.9	0.00∗
Light	104.6 ± 36.1∗∗∗	54–195	122.7 ± 22.2	66–159	33.4	0.00∗
Moderate	154.9 ± 50.1∗∗∗	85–263	210.8 ± 51.1	139–304	16.4	0.00∗
Total MVPA	165.9 ± 58.7∗∗	86–286	218.3 ± 65.6	143–358	13.7	0.01∗

Note: M: mean; SD: standard deviation. *P ≤ 0.05.

**P ≥ 0.01.

***P ≥ 0.001.
